# Impact of COPD on Outcomes After Pulmonary Metastasectomy: A Propensity-Score Matched Analysis †

**DOI:** 10.3390/cancers18142353

**Published:** 2026-07-21

**Authors:** Konstantinos Grapatsas, Fabian Doerr, Roemer van Wijk, Thomas Bergmann, Ilias Arfanis, Viktor Grünwald, Sebastian Bauer, Stephan Lang, Stefan Kasper, Boris Hadaschik, Dirk Schadendorf, Ulf Neumann, Christian Taube, Martin Schuler, Servet Bölükbas, Natalie Baldes

**Affiliations:** 1 Department of Thoracic Surgery, West German Cancer Center, Medical Faculty, University Hospital Essen, Ruhrlandklinik, Tueschner Weg 40, 45239 Essen, Germanyroemer.vanwijk@rlk.uk-essen.de (R.v.W.); thomas.bergmann@rlk.uk-essen.de (T.B.); ilias.arfanis@rlk.uk-essen.de (I.A.); servet.boeluekbas@rlk.uk-essen.de (S.B.); natalie.baldes@rlk.uk-essen.de (N.B.); 2 Interdisciplinary GU Oncology, Clinic for Urology and Clinic for Medical Oncology, West German Cancer Center Essen, University Hospital Essen, 45147 Essen, Germany; viktor.gruenwald@uk-essen.de; 3 Department of Medical Oncology, Sarcoma Center, West German Cancer Center, Medical Faculty, University Hospital Essen, Hufelandstr. 55, 45147 Essen, Germany; sebastian.bauer@uk-essen.de; 4 Department of Otorhinolaryngology, Medical Faculty, University Hospital Essen, Hufelandstr. 55, 45147 Essen, Germany; stephan.lang@uk-essen.de; 5 Department of Medical Oncology, West German Cancer Center, Medical Faculty, University Hospital Essen, Hufelandstr. 55, 45147 Essen, Germany; stefan.kasper-virchow@uk-essen.de (S.K.); martin.schuler@uk-essen.de (M.S.); 6 Department of Urology, University Hospital Essen, Hufelandstr. 55, 45147 Essen, Germany; boris.hadaschik@uk-essen.de; 7 Department of Dermatology, University Hospital Essen, Hufelandstr. 55, 45147 Essen, Germany; dirk.schadendorf@uk-essen.de; 8 General, Visceral and Transplantation Surgery, University Hospital Essen, Hufelandstr. 55, 45147 Essen, Germany; ulf.neumann@uk-essen.de; 9 Department of Pneumology, Medical Faculty, University Hospital Essen, Hufelandstr. 55, 45147 Essen, Germany; christian.taube@rlk.uk-essen.de

**Keywords:** chronic obstructive pulmonary disease, pulmonary metastasectomy, 30-day-mortality, postoperative complications, thoracoscopy, thoracotomy, thoracic surgery

## Abstract

Background: Pulmonary metastasectomy (PM) is an established component of multimodal oncological treatment, but its perioperative safety and long-term outcomes in patients with chronic obstructive pulmonary disease (COPD) remain insufficiently defined. We aimed to evaluate the impact of COPD on perioperative morbidity, mortality and long-term survival after PM. Methods: We retrospectively analysed 692 patients who underwent PM with curative intent. COPD severity was graded using the GOLD spirometric classification. Patients were classified according to their postbronchodilator FEV1/FVC ratio into a COPD group (<0.70) and a control group (≥0.70). Groups were compared regarding clinical and surgical characteristics, postoperative morbidity and mortality, and overall survival (Kaplan–Meier, log-rank). Independent predictors of morbidity and survival were identified via logistic and Cox regression, and a 1:1 propensity-score-matched analysis was performed to account for baseline imbalances. Results: Among 9684 patients, 692 (7.1%) patients underwent PM, of whom 171 (24.7%) had COPD. They were more frequently smokers and had a higher comorbidity burden and worse performance status and, as expected, significantly lower lung function. The main clinically relevant finding was a significantly higher rate of persistent air leak in patients with COPD (8.8% vs. 2.3%, *p* < 0.001). Overall morbidity and 30-day mortality, however, did not differ. With a median follow-up of 76.7 months, survival was similar between groups (3-year survival 76.5% vs. 78.4%, *p* = 0.495) and was unaffected by GOLD severity. Independent predictors of worse survival were preoperative systemic therapy (HR 2.18), repeat metastasectomy (HR 1.60) and increasing age (HR 1.02 per year). After propensity matching, survival remained comparable (HR 1.05, *p* = 0.835). Conclusions: Among carefully selected patients, COPD was not associated with increased mortality or reduced survival after PM, although persistent air leak was significantly more frequent. COPD alone should not, by itself, be considered a contraindication to curative-intent surgery in appropriately selected patients, and these findings should not be generalized to patients with severe or end-stage COPD.

## Simple Summary

Chronic obstructive pulmonary disease (COPD) is a frequent comorbidity that is often perceived as a relative contraindication to thoracic surgery. Pulmonary metastasectomy, the surgical removal of lung metastases, can prolong survival in selected patients with metastatic cancer. However, its safety in patients with coexisting COPD has not been well established. In this retrospective study, we compared 171 patients with COPD and 521 without COPD who underwent pulmonary metastasectomy at our institution. Postoperative mortality and long-term survival were comparable between the two groups, and this finding persisted across all disease-severity grades and after propensity-score matching. The principal difference was a higher rate of prolonged air leak in patients with COPD. These results indicate that COPD alone should not preclude carefully selected patients from undergoing curative-intent pulmonary metastasectomy.

## 1. Introduction

Pulmonary metastasectomy (PM) is the surgical resection of lung metastases that most commonly arise from extrathoracic primary tumours. Over the past decades, it has evolved into a well-established component of the multimodal treatment of selected oncological patients, particularly when the metastatic disease is confined to the lungs, the primary tumour is controlled, and complete (R0) resection is considered achievable [1,2,3]. Because most metastases are peripheral and amenable to parenchyma-sparing resection, PM is frequently performed as a lung-sparing procedure, preserving pulmonary function and broadening the range of patients who can be considered for surgery. Nevertheless, its perioperative safety and long-term outcomes of PM in patients with relevant pulmonary comorbidity, such as chronic obstructive pulmonary disease (COPD), remain insufficiently defined.

Chronic obstructive pulmonary disease is a common, progressive disorder characterized by persistent airflow limitation and represents a major global health burden, affecting more than 300 million people worldwide. It encompasses a spectrum of conditions, including chronic bronchitis and emphysema, that lead to airflow obstruction, dyspnea, and a range of systemic effects [4]. Owing largely to shared risk factors, most importantly tobacco smoking, COPD frequently coexists with malignancy. Beyond non-small cell lung cancer (NSCLC), it is associated with a variety of extrapulmonary tumours and with a substantial comorbidity burden, including cardiovascular disease, cachexia, and gastrointestinal disorders [4]. In the surgical setting, COPD reduces physiological reserve and increases susceptibility to infection, and it has consistently been linked to higher postoperative morbidity and mortality after major thoracic procedures such as anatomical lung resection for NSCLC [5,6].

For these reasons, COPD is widely regarded as a limiting factor in thoracic surgical decision-making, and patients with impaired lung function are often considered higher-risk surgical candidates [7]. However, the evidence concerning PM specifically in COPD patients is scarce and conflicting. Moreover, most of the available data derive from anatomical resections for NSCLC rather than from the predominantly parenchyma-sparing resections used in PM, which limits their applicability to selection [6,7,8,9,10,11,12,13,14,15].

The present study was designed to investigate the safety and survival outcomes of PM in patients with COPD. Given the challenges of performing thoracic surgery in patients with compromised lung function, the primary aim was to determine whether PM can be performed safely in this population and whether postoperative morbidity and survival after PM differ between patients with and without COPD. The secondary aim was to characterize the comorbidities and postoperative complications of COPD patients undergoing PM, including an analysis stratified by COPD severity.

## 2. Materials and Methods

### 2.1. Study Design

This was a retrospective analysis of prospectively collected data from a single-institution thoracic surgery database. Part of this cohort overlaps with a previously reported institutional series that addressed a different research question of the development of a postoperative-morbidity prediction score. The present study extends that database and focuses specifically on the impact of COPD on perioperative outcomes and survival [15].

### 2.2. Data Collection

All patient data were entered into the database at the time of treatment and were subsequently anonymized prior to analysis. After anonymization, no patient could be re-identified, in line with applicable data-protection regulations.

### 2.3. Ethics Approval

Ethical approval for the retrospective extraction and analysis of data on patients undergoing PM was granted by the local institutional ethics committee, as part of a broader approved study on pulmonary metastases (approval no. 23-11273-BO; 22 May 2023). All study procedures were conducted in accordance with the principles of the Declaration of Helsinki. Informed consent was waived due to the retrospective design of the study.

### 2.4. Inclusion and Exclusion Criteria

Patients who underwent PM at our institution between 2015 and January 2023 were included. Eligible patients were those who underwent PM with curative intent as part of an oncological treatment strategy approved preoperatively by a multidisciplinary tumour board, ensuring that all procedures were part of a clinically justified treatment plan. The following exclusion criteria were applied: age under 18 years, procedures performed for diagnostic or palliative purposes only and resection of recurrent lung lesions originating from a primary lung cancer.

### 2.5. Definition of Study Groups

Patients were divided into two groups according to their postbronchodilator FEV1/FVC ratio. The control group comprised patients with preserved lung function (FEV1/FVC ≥ 0.70), and the COPD group comprised patients with airflow obstruction (FEV1/FVC < 0.70). Patients with a documented history of asthma or asthma–COPD over-lap, identified from the medical records and preoperative clinical evaluation, were not classified as having COPD. As this was a retrospective study, bronchodilator reversibility testing was not performed according to a standardized protocol. The distinction between COPD and asthma therefore relied on the pre-existing clinical diagnosis documented in the patients’ medical history.

### 2.6. Classification of COPD Severity

Within the COPD group, the severity of airflow limitation was graded according to the GOLD spirometric classification, based on the postbronchodilator FEV1 (% predicted): GOLD 1 (mild), FEV1 ≥ 80%; GOLD 2 (moderate), 50% ≤ FEV1 < 80%; GOLD 3 (severe), 30% ≤ FEV1 < 50%; and GOLD 4 (very severe), FEV1 < 30%. For the analysis of outcomes by severity, the COPD group was further dichotomized into a mild-to-moderate subgroup (GOLD 1–2) and a severe-to-very-severe subgroup (GOLD 3–4) [8,9]. The multidimensional GOLD assessment (symptom burden and exacerbation history or ABE groups) was not applied, as this study focused on pulmonary functional reserve as a perioperative risk factor.

### 2.7. Definitions of Comorbidity and Postoperative Morbidity

Patients aged 70 years or older were classified as elderly, and obesity was defined as a body mass index (BMI) > 30 kg/m^2^. Documented comorbidities included conditions requiring regular pharmacological treatment, such as arterial hypertension, diabetes mellitus, and cardiovascular disease. All comorbidities were recorded preoperatively [10]. Postoperative morbidity was defined as any new medical condition requiring treatment within 30 days of surgery. A persistent air leak was defined as an air leak lasting more than five days postoperatively [10,11].

### 2.8. Definitions of Disease-Free Interval, Disease-Free Survival, Mortality, Overall Survival and Follow-Up

The disease-free interval (DFI) was defined as the interval between resection or definitive treatment of the primary tumour and the first detection of pulmonary metastases. A DFI of zero was assigned to patients with synchronous metastases.

Disease-free survival (DFS) was defined as the time from PM to the first radiological evidence of recurrence.

Mortality was defined as death from any cause and was assessed as 30-day and 90-day postoperative mortality. Overall survival (OS) was calculated from the date of lung surgery to death from any cause or to the last follow-up. Vital status was obtained from the institutional database.

### 2.9. Preoperative Therapy, Surgical Approach and Extent of Resection

Preoperative systemic therapy was defined as any oncological treatment administered within eight weeks before PM and comprised chemotherapy, chemotherapy plus radiotherapy, or immunotherapy-based regimens. Treatments that did not fit these categories were classified as other/unspecified, and cases without available information were recorded as unknown.

All resections, including primary PM and repeated pulmonary metastasectomy (RPM), were performed under general anaesthesia with curative intent. Identical indication criteria were applied to PM and RPM. A double-lumen endotracheal tube was used in all cases. Procedures were carried out under general anaesthesia, using either a thoracotomy or a thoracoscopic (VATS) approach depending on tumour location and number. Resections comprised wedge resection, segmentectomy, lobectomy, or pneumonectomy, with the approach and extent of resection determined by the location and number of metastases. The number of resected metastases was categorized as single, 2–5, or multiple (>5). Lymphadenectomy was performed as lymph node sampling at the surgeon’s discretion. RPM was defined as a metastasectomy performed in a patient with a history of previous parenchyma-resecting lung surgery. Because this history was established at the time of the index operation, from which overall survival was measured, RPM was analysed as a fixed baseline covariate. Major resection was defined as lobectomy, bilobectomy or pneumonectomy, whereas wedge resection and segmentectomy were classified as minor resections.

### 2.10. Statistical Analysis

Categorical variables were compared using the χ^2^ test or Fisher’s exact test, as appropriate. Continuous variables are presented as median (range) and were compared using the Mann–Whitney U test. Overall survival was estimated by the Kaplan–Meier method, and differences between groups were assessed with the log-rank test. Follow-up was estimated using the reverse Kaplan–Meier method.

Independent predictors of overall survival were identified by Cox proportional-hazards regression, and independent predictors of postoperative morbidity by logistic regression. For both models, variables with a *p* value < 0.05 in univariable analysis were entered into the multivariable model. As COPD was the principal exposure of interest, it was forced into the multivariable models irrespective of its univariate significance. Because the presence of COPD and GOLD severity represent the same underlying condition, they were entered into separate multivariable models to avoid collinearity. Results are reported as hazard ratios (HR) or odds ratios (OR) with 95% confidence intervals. Candidate variables for the survival model also included primary tumour histology (sarcoma, colorectal, renal cell carcinoma, melanoma, other).

To account for baseline differences between groups, a propensity-score-matched analysis was additionally performed. A propensity score for the presence of COPD was estimated by multivariable logistic regression including age, gender, obesity, ASA class, ECOG performance status, presence of any comorbidity, arterial hypertension, cardiovascular disease, chronic kidney disease, smoking status, primary tumour histology, number of resected metastases, preoperative systemic therapy, and extent of resection. Patients were matched 1:1 by nearest-neighbour matching without replacement, using a caliper of 0.2 times the standard deviation of the logit of the propensity score. Balance between the matched groups was assessed using standardized mean differences, with an absolute value <0.10 considered to indicate adequate balance.

All analyses were performed using SPSS version 22.0 (IBM SPSS Inc., Chicago, IL, USA). A two-sided *p* value < 0.05 was considered statistically significant.

## 3. Results

### 3.1. Formation of the Study Population

**Figure 1:**
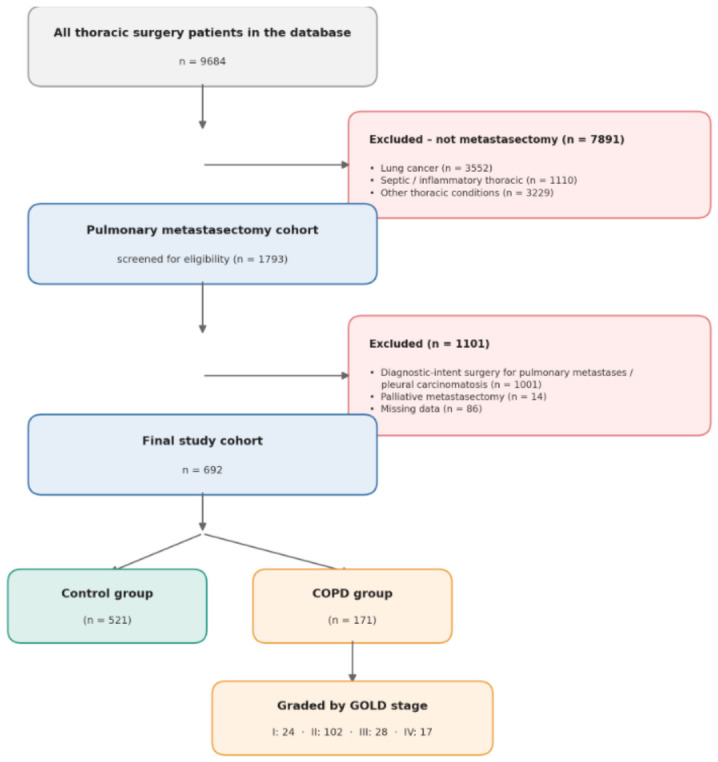
Patients (24.7%) were assigned to the COPD group, while 521 patients (75.3%) constituted the control group. Most COPD patients were classified as GOLD 2 (59.6%) or GOLD 1 (14.0%), with smaller proportions in GOLD 3 (16.4%) and GOLD 4 (9.9%). The distribution of the COPD group in its subgroups is demonstrated in Table 1.

Among 9684 patients receiving thoracic surgery at the study time, 692 (7.1%) patients underwent PM with curative intent and formed the final study cohort for the analysis of PM outcomes (Figure 1).

**Table 1:** Distribution of COPD severity (GOLD grade) among COPD patients undergoing pulmonary metastasectomy (*n* = 171).

COPD GOLD Stage	COPD Group (*n* = 171)
I	24 (14.0%)
II	102 (59.6%)
III	28 (16.4%)
IV	17 (9.9%)

### 3.2. Clinical and Surgical Characteristics of the Study Population

Patients in the COPD group more frequently had an ECOG performance status ≥1 (22.2% vs. 14.6%, *p* = 0.013) and a higher comorbidity burden, with at least one documented comorbidity in 90.6% versus 81.6% of controls (*p* = 0.007) and arterial hypertension in 50.9% versus 40.7% (*p* = 0.025). As expected, smoking history differed markedly between the groups (*p* < 0.001), with a higher proportion of current and former smokers in the COPD group. In contrast, gender, age, body mass index and cardiovascular disease did not differ significantly between the groups. As expected, pulmonary function was significantly lower in the COPD group, with a lower FEV1/FVC ratio (0.59 ± 0.10 vs. 0.80 ± 0.06, *p* < 0.001), FEV1 (1.71 ± 0.68 vs. 2.51 ± 0.84 L, *p* < 0.001), FEV1% predicted (59.6 ± 18.9 vs. 86.3 ± 20.3, *p* < 0.001) and DLCO % predicted (53.7 ± 17.3 vs. 70.2 ± 16.4, *p* < 0.001).

Regarding surgical characteristics, lung-sparing wedge resections and segmentectomies predominated in both groups, and the type of resection did not reach statistical significance (*p* = 0.051). Lymphadenectomy was performed more frequently in the COPD group (56.7% vs. 46.1%, *p* = 0.019), and RPM was also more common in this group (17.0% vs. 8.6%, *p* = 0.004). The surgical approach, the number of resected metastases and the use of preoperative therapy did not differ significantly between the groups. The clinical and surgical characteristics of the study population are displayed in Table 2 and Table 3.

### 3.3. Histology of the Primary Tumours

No statistically significant difference was observed in the histological types of the primary tumours between the two groups (*p* = 0.757). Metastases originating from sarcomas were the most frequently resected in both groups (35.3% vs. 37.4%), followed by colorectal and renal cell carcinoma. The histological types of the primary tumours are shown in Table 4. When entered into the Cox model, primary tumour histology was not associated with overall survival (overall log-rank *p* = 0.863).

### 3.4. Postoperative Morbidity and Complications

The overall rate of postoperative complications was slightly higher in the COPD group (19.3% vs. 14.6%), but this difference was not statistically significant (*p* = 0.148). The only complication that differed significantly between the groups was a persistent air leak lasting more than five days, which was substantially more frequent in the COPD group (8.8% vs. 2.3%, *p* < 0.001). Postoperative morbidity is detailed in Table 5. When outcomes were stratified by the extent of resection, the higher rate of prolonged air leak in patients with COPD persisted after both major resection (7.6% vs. 0.7%) and wedge/sublobar resection (9.5% vs. 2.9%), whereas overall morbidity and 30-day mortality remained low in both strata.

### 3.5. Postoperative Mortality—30-Day and 90-Day Mortality

Mortality was low in the overall study population. No 30-day mortality was observed in the COPD group (0%, *n* = 0), compared with 1.7% in the control group (*p* = 0.122). The 90-day mortality was also comparable between the groups (3.5% vs. 2.3%, *p* = 0.409). Postoperative mortality rates are detailed in Table 5.

### 3.6. Overall Survival

**Figure 2:**
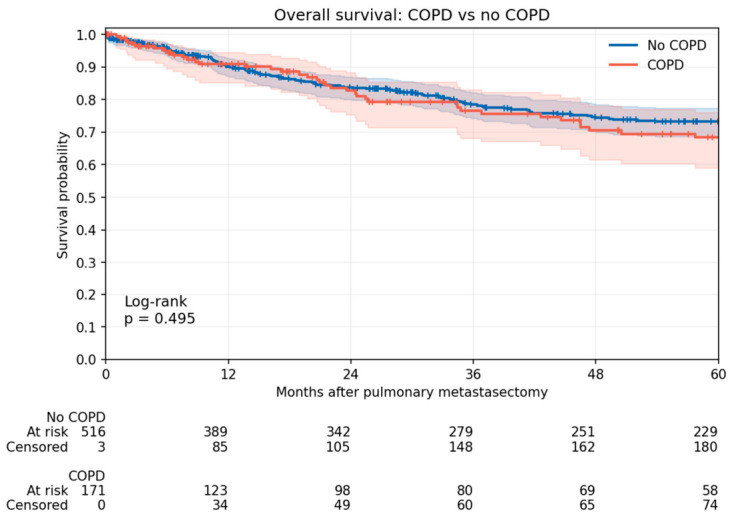
Overall survival of COPD and control (no COPD) groups.

The median follow-up of the cohort was 76.7 months. Survival was comparable between the groups throughout follow-up. The 1-, 2- and 3-year survival rates were 90.9%, 82.7% and 76.5% in the COPD group versus 90.2%, 83.8% and 78.4% in the control group, with no significant difference in the log-rank test (*p* = 0.495). The Kaplan–Meier curves of the two groups are shown in Figure 2.

### 3.7. Survival and Baseline Characteristics by COPD Severity

**Figure 3:**
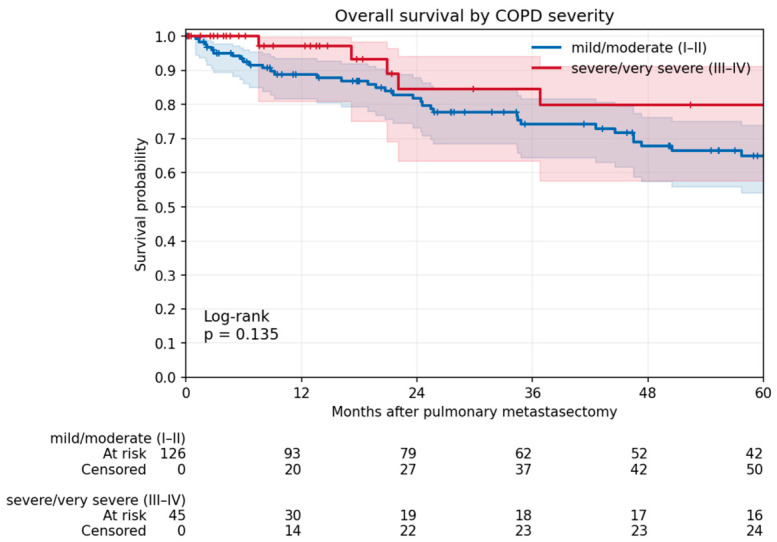
Overall survival among COPD severity groups.

When the COPD group was dichotomized into mild/moderate (GOLD 1–2) and severe/very severe (GOLD 3–4) subgroups, no significant difference in survival was observed between the two subgroups in the log-rank test (*p* = 0.135), nor across the four individual GOLD grades (*p* = 0.254). The Kaplan–Meier curves for the COPD subgroups are demonstrated in Figure 3.

To clarify why patients with severe/very severe COPD did not show worse survival, baseline oncological and surgical characteristics were compared between the mild/moderate (GOLD 1–2) and severe/very severe (GOLD 3–4) subgroups (Table 6). Patients with GOLD 3–4 disease underwent major anatomical resection significantly less often (15.6% vs. 46.8%, *p* < 0.001) and received preoperative systemic therapy less frequently (6.7% vs. 23.0%, *p* = 0.015), with a tendency toward more minimally invasive procedures (VATS 57.8% vs. 39.7%, *p* = 0.054), whereas tumour histology, number of metastases, laterality and repeat metastasectomy did not differ significantly. This indicates that the more advanced COPD subgroup was managed with a more conservative, parenchyma-sparing surgical strategy and had less aggressive oncological features, which most likely explains the absence of worse survival in this subgroup rather than any protective effect of COPD severity.

### 3.8. Independent Factors for Postoperative Morbidity

In the logistic regression analysis, an ECOG performance status ≥ 1 (OR 2.09, 95% CI 1.28–3.41, *p* = 0.003), RPM (OR 1.88, 95% CI 1.06–3.35, *p* = 0.031) and the VATS approach (OR 0.58, 95% CI 0.37–0.89, *p* = 0.013; protective) remained independent predictors of postoperative morbidity. The presence of COPD itself was not associated with postoperative morbidity (*p* = 0.144), although increasing GOLD severity was associated with morbidity in the univariable analysis (OR 1.20 per grade, *p* = 0.045). The results are shown in Table 7.

### 3.9. Independent Factors for Survival

In the Cox regression analysis, preoperative systemic therapy (HR 2.18, 95% CI 1.49–3.20, *p* < 0.001), the need for repeated resection of lung metastases (HR 1.60, 95% CI 1.03–2.50, *p* = 0.036) and increasing age (HR 1.02 per year, *p* = 0.003) were independent predictors of worse overall survival. Neither the presence of COPD (HR 1.13, *p* = 0.496) nor its GOLD severity (HR 1.01 per grade, *p* = 0.917) was associated with survival. The results are shown in Table 8. Primary tumour histology was also tested and showed no association with survival and was therefore not retained in the multivariable model.

### 3.10. Propensity Score-Matched Analysis

**Figure 4:**
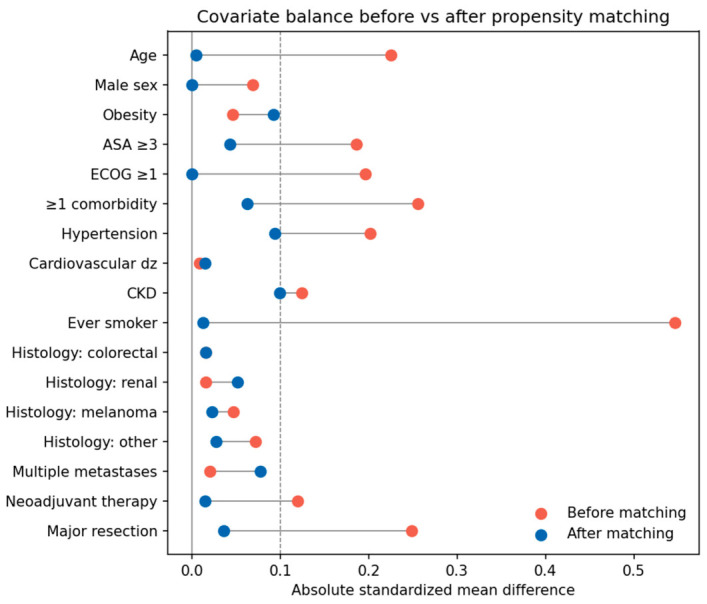
Love plot demonstrating improved covariate balance after propensity-score matching.

After 1:1 propensity-score matching using a propensity model, 170 COPD patients were matched to 170 controls (*n* = 340), yielding well-balanced groups (all standardized mean differences ≤ 0.10, Table 9, Figure 3). In the matched cohort, overall survival did not differ between COPD and control patients (HR 1.05, 95% CI 0.67–1.65, *p* = 0.835; log-rank *p* = 0.836, Table 10), confirming the results of the overall analysis (Figure 4).

## 4. Discussion

The main finding of our study is that PM in patients with COPD is safe, is not associated with increased mortality, and was associated with long-term survival comparable to that of patients without COPD. Importantly, the presence of COPD neither shortened nor improved survival, and this lack of effect persisted after propensity-score matching, indicating that it was not driven by imbalances in baseline characteristics. To our knowledge, this is the first study designed specifically to evaluate the impact of COPD on outcomes after PM.

As expected from the shared risk factor of tobacco exposure, patients in the COPD group were significantly more often current or former smokers. They also presented with a higher comorbidity burden, including arterial hypertension, and more frequently had an impaired performance status (ECOG ≥ 1). Such a comorbidity burden is characteristic of COPD patients, in whom cardiovascular disease and malignancies are particularly frequent [4,13]. Although patients with COPD had significantly lower FEV1, FEV1% predicted and DLCO, this did not translate into higher perioperative morbidity or worse long-term survival. In addition, most COPD patients had mild-to-moderate (GOLD 1–2) disease, reflecting careful preoperative selection. In terms of resection strategy, both groups were managed predominantly with a lung-sparing approach.

It is generally accepted that patients with comorbidities, including COPD, should not be excluded from PM with curative intent [1,15,16,17,18,19]. Nevertheless, the negative impact of COPD on surgical outcomes, particularly after lung resection for NSCLC, presents a dilemma in treating these patients surgically [14]. For NSCLC, COPD has long been viewed as a factor that may preclude or complicate major anatomical lung resection. In a large retrospective study of 1239 patients, Licker et al. reported that an FEV1 < 60% is a significant risk factor for perioperative mortality and postoperative complications [20], and Sekine et al. found worse pulmonary complications and overall survival in COPD patients [21]. In contrast, Senbaklavaci found no significant increase in postoperative complications among elderly COPD patients undergoing lobar resection [14]. Research on postoperative morbidity following PM specifically in COPD patients is limited. Hassan et al. and Rodríguez-Fuster et al. identified COPD or pre-existing respiratory disease as a risk factor for postoperative complications [17,22], whereas Sponholz et al. did not [23]. One possible explanation for the favorable outcomes observed in our COPD patients is the predominantly parenchyma-preserving surgical strategy adopted in PM, in contrast to the anatomical lobar resections often required for NSCLC, which may protect this population from the higher morbidity reported after major anatomical resections [5,6].

In our cohort, the overall postoperative complication rate did not differ significantly between the COPD and control groups. The single exception was a persistent air leak lasting more than five days, which—as would be expected in patients with airflow obstruction and emphysematous parenchyma—was significantly more frequent in the COPD group (8.8% vs. 2.3%). Crucially, this higher rate of air leak did not translate into increased mortality. Increasing GOLD severity, an ECOG performance status ≥ 1, surgical approach and RPM emerged as factors associated with increased postoperative morbidity. These associations are clinically plausible. Greater airflow limitation and poorer performance status reflect reduced physiological reserve. The minimally invasive VATS approach is consistently associated with fewer complications, and repeat surgery in a previously operated hemithorax is technically more demanding [11].

At our institution, prevention of postoperative air leak follows a systematic but individualized approach, tailored to the operative technique and to the patient’s pulmonary reserve. The surgical technique is tailored to the operative approach and to the patient’s pulmonary reserve. In VATS procedures, pulmonary metastases are generally resected using endoscopic stapling devices. In open metastasectomy and particularly when multiple metastases are resected in patients with adequate pulmonary function, the lung parenchyma is frequently closed with sutures in order to minimize the loss of functioning lung tissue. In patients with severe COPD (GOLD 3–4), buttressed stapling devices, analogous to those used in lung-volume-reduction surgery, may be employed to reinforce the staple line in emphysematous parenchyma. In all cases, the use of adjunctive measures such as fibrin sealants and buttressing materials over the staple line remains at the discretion of the operating surgeon [1].

The perioperative impact of COPD after lung surgery is closely related to the volume of lung parenchyma removed. Because PM is predominantly parenchyma-sparing, with wedge resection being the most frequent procedure, the limited resection volume likely mitigates the functional and perioperative consequences of COPD, and the comparable survival between groups may therefore partly reflect this conservative resection strategy rather than an absence of COPD-related risk. Consistent with this, patients with severe/very severe COPD underwent major anatomical resection significantly less often than those with mild/moderate disease. In a subgroup analysis stratified by extent of resection, the higher rate of prolonged air leak in patients with COPD persisted after both major resection (7.6% vs. 0.7%) and wedge/sublobar resection (9.5% vs. 2.9%), whereas overall morbidity and mortality remained low in both strata. This suggests that the parenchyma-sparing approach helps contain the overall perioperative risk, even though the COPD-related predisposition to air leak persists irrespective of resection extent.

In addition, it is important to distinguish perioperative pulmonary risk from long-term oncological survival, as these outcomes are governed by different determinants. COPD is biologically and clinically expected to affect postoperative pulmonary complications, particularly prolonged air leak, respiratory morbidity and tolerance of lung resection, as reflected by the higher air-leak rate observed in our cohort. In contrast, OS after PM is largely dominated by oncological factors such as tumour biology, disease burden, DFI and the indication for RPM. Accordingly, the absence of a significant difference in OS does not imply that COPD is irrelevant to surgical risk. Rather, its perioperative impact may be masked in survival analyses by these stronger tumour-related determinants.

With respect to survival, COPD and its associated comorbidities have been reported to influence prognosis adversely in lung cancer [24,25,26]. Sekine et al. and Hassan et al. identified COPD as an adverse factor for survival after lung resection and PM, respectively [17,21]. However, no such effect was seen in Senbaklavaci’s series [14]. In our study, neither the presence of COPD nor its GOLD severity was associated with overall survival in univariable or multivariable analysis. Instead, increasing age, preoperative systemic therapy and RPM were independent predictors of worse survival. The association with age is expected, as advancing age is accompanied by a greater comorbidity burden and higher competing mortality. The association with preoperative systemic therapy most likely reflects a higher underlying oncological burden in these patients rather than a detrimental effect of the treatment itself, and should not be misinterpreted as such. Similarly, the adverse prognostic effect of RPM probably reflects the recurrent and biologically more aggressive nature of the metastatic disease, rather than a harmful effect of the operation per se [27,28,29,30,31]. More broadly, these oncological predictors—reflecting tumour biology, disease burden, treatment resistance and recurrence pattern—are considerably stronger determinants of long-term outcome than pulmonary comorbidity in this stage IV surgical population. Any prognostic contribution of COPD may therefore be statistically diluted or masked by these dominant oncological factors, which may partly explain the absence of an independent association between COPD and OS. The apparently more favourable survival observed in the severe/very severe (GOLD 3–4) subgroup should not be interpreted as a protective effect of advanced COPD. As shown in the stratified analysis, these patients underwent major anatomical resection significantly less often and received preoperative systemic therapy less frequently, reflecting a more conservative, parenchyma-sparing surgical strategy and less aggressive oncological features. Together with the small number of GOLD 3–4 patients and the resulting limited statistical power, this indicates that the survival pattern in this subgroup is most likely driven by selection and indication bias rather than by COPD severity itself.

To address the numerical imbalance between groups (171 vs. 521 patients), we performed a propensity-score-matched analysis. After 1:1 matching, survival remained comparable between COPD and control patients, further supporting the central finding that COPD was not associated with worse outcomes.

In addressing the dilemma of treating patients with COPD, we believe these individuals should not be excluded from a potentially curative PM. We recommend that patients with COPD undergo thorough preoperative evaluation within an oncological framework approved by a multidisciplinary tumour board. Where appropriate, optimizing antiobstructive therapy and implementing neoadjuvant prehabilitation, together with a parenchyma-sparing surgical strategy and, when feasible, a minimally invasive VATS approach, can further enhance outcomes for this challenging population [14,26].

### Limitations

Our study has several limitations. First, it is a retrospective, single-center analysis and is therefore inherently subject to selection bias. Patients with diffuse pulmonary metastases or poor general condition or the most severe pulmonary impairment may not have been offered surgery and were thus not included, and neither the decision to operate nor the surgical approach was randomized. Second, although the COPD and control groups were numerically unbalanced, this was addressed by propensity-score matching, which confirmed the main findings. Nevertheless, propensity-score matching can only balance measured covariates and cannot adjust for unmeasured oncological selection fac-tors, such as systemic treatment response, molecular subtype, pace of disease progression, tumour doubling time, extrapulmonary disease control, or the multidisciplinary decision-making process leading to surgery, so a residual selection bias cannot be excluded. Furthermore, the absence of statistically significant differences should not be interpreted as evidence of equivalence. A formal equivalence or non-inferiority design would be required to establish comparable safety. However, because smoking status was incompletely recorded (unknown in 54.3% of controls and 29.2% of COPD patients), residual confounding cannot be excluded, and the propensity-score analysis that incorporated smoking should be interpreted with caution. Because the proportion of missing smoking data differed between groups, being substantially higher in the control group, and because smoking is associated with both COPD and oncological outcomes, this differential missingness may have biased the between-group comparison of smoking exposure and could not be fully resolved by propensity-score adjustment. The higher proportion of recorded smokers in the COPD group should therefore be interpreted with caution, and residual confounding by smoking intensity (e.g., pack–years) cannot be excluded. Moreover, COPD was defined solely on the basis of spirometric airflow limitation (postbronchodilator FEV1/FVC < 0.70), without incorporating the current GOLD ABE classification, symptom burden, exacerbation history or radiological findings. In addition, the majority of patients had mild-to-moderate (GOLD 1–2) disease, and only 45 patients had severe or very severe (GOLD 3–4) COPD. Statistical power for this subgroup was therefore limited, and the results cannot be extrapolated to patients with severe or end-stage COPD. Moreover, because no non-operated COPD control group was available, our study cannot establish a causal oncological benefit of PM and can only show that perioperative and survival outcomes were comparable between operated patients with and without COPD. Lastly, overall survival may be a relatively insensitive endpoint for capturing the clinical impact of COPD in this setting, as it is strongly influenced by cancer-related factors and subsequent systemic therapy. COPD-related effects might be better reflected by endpoints such as postoperative respiratory morbidity, postoperative decline in pulmonary function, readmission, non-cancer mortality, or treatment tolerance after recurrence, which were not systematically available in our dataset and warrant evaluation in future studies.

## 5. Conclusions

In one of the first studies to specifically evaluate the impact of COPD on outcomes after PM, COPD was not statistically associated with increased mortality or reduced long-term survival among carefully selected patients undergoing curative-intent metastasectomy, even after propensity-score matching, although prolonged air leak was significantly more frequent in patients with COPD. Given the retrospective design and the possibility of selection bias, patients with the most severe pulmonary impairment may not have been referred for surgery. These findings should not be interpreted as evidence of equivalence or absence of risk and cannot be generalized to all patients with COPD, particularly those with severe or end-stage disease. Nevertheless, within these limits, COPD alone should not be considered an absolute contraindication to curative-intent metastasectomy in appropriately selected and optimized patients.

## Figures and Tables

**Table 2:** Clinical characteristics of patients undergoing pulmonary metastasectomy in control and COPD groups.

Variable	Control *n* = 521 (%)	COPD *n* = 171 (%)	*p*-Value
Gender			0.474
Male	310 (59.5)	96 (56.1)	
Female	211 (40.5)	75 (43.9)	
Age			0.130
≤70 years	379 (72.7)	128 (74.9)	
>70 years (elderly)	142 (27.3)	43 (25.1)	
BMI			0.709
≤30	446 (85.6)	149 (87.1)	
>30 (obesity)	75 (14.4)	22 (12.9)	
ASA score			0.160
ASA 1	413 (79.3)	123 (71.9)	
ASA 2	38 (7.3)	13 (7.6)	
ASA 3	63 (12.1)	31 (18.1)	
ASA 4	7 (1.3)	4 (2.3)	
ECOG performance status			0.013
ECOG 0	445 (85.4)	133 (77.8)	
ECOG 1	58 (11.1)	32 (18.7)	
ECOG 2	16 (3.1)	3 (1.8)	
ECOG ≥ 3	2 (0.4)	3 (1.8)	
Pulmonary function			
FEV1, L (mean ± SD)	2.51 ± 0.84	1.71 ± 0.68	<0.001
FEV1, % predicted (mean ± SD)	86.3 ± 20.3	59.6 ± 18.9	<0.001
DLCO, % predicted (mean ± SD)	70.2 ± 16.4	53.7 ± 17.3	<0.001
FEV1/FVC ratio (mean ± SD)	0.80 ± 0.06	0.59 ± 0.10	<0.001
At least one comorbidity	425 (81.6)	155 (90.6)	0.007
Art. Hypertension	212 (40.7)	87 (50.9)	0.025
Cardiovascular disease	102 (19.6)	33 (19.3)	1.000
Chronic kidney disease	24 (4.6)	4 (2.3)	0.263
Smoking status			<0.001
Current smoker	98 (18.8)	49 (28.7)	
Former smoker	105 (20.2)	63 (36.8)	
Never smoker	35 (6.7)	9 (5.3)	
	283 (54.3)	50 (29.2)	

**Table 3:** Surgical characteristics in patients undergoing pulmonary metastasectomy in control and COPD groups.

Variable	Control *n* = 521 (%)	COPD *n* = 171 (%)	*p*-Value
DFI (median, range)	24 (0–120)	23 (0–119)	0.9
DFS	11 (1.5–96)	12 (2–98)	0.9
Side of the operation			0.054
Unilateral	480 (92.1%)	165 (96.5%)	
Bilateral	41 (7.9%)	6 (3.5%)	
Type of resection			0.051
Wedge resection	361 (69.3)	100 (58.5)	
Segmentectomy	20 (3.8)	5 (2.9)	
Lobectomy	127 (24.4)	61 (35.7)	
Bilobectomy	8 (1.5)	2 (1.2)	
Pneumonectomy	5 (1.0)	3 (1.8)	
Surgical approach			0.501
Open thoracotomy	272 (52.2)	95 (55.6)	
VATS	249 (47.8)	76 (44.4)	
Lymphadenectomy			0.019
Not performed	278 (53.4)	73 (42.7)	
Performed	240 (46.1)	97 (56.7)	
unknown	3 (0.6)	1 (0.6)	
Metastases resected			0.899
Single	93 (17.9)	32 (18.7)	
2–5 metastases	332 (63.7)	110 (64.3)	
R0 resection	515 (98.8%)	167 (97.7%)	0.1
Mediastinal lymph node metastases	4 (0.8%)	2 (1.2%)	0.9
Multiple	96 (18.4)	29 (17.0)	
Procedure			0.004
Single PM	476 (91.4)	142 (83.0)	
RPM (repeat)	45 (8.6)	29 (17.0)	
Preoperative therapy			0.190
None	441 (84.6)	138 (80.7)	
Chemotherapy	29 (5.6)	10 (5.8)	
Chemotherapy + radiotherapy	43 (8.3)	19 (11.1)	
Immunotherapy-based	0 (0.0)	0 (0.0)	
Other/unspecified	2 (0.4)	3 (1.8)	
unknown	6 (1.2)	1 (0.6)	

**Table 4:** Primary tumour histology of the study population.

Primary Tumour	Control *n* = 521 (%)	COPD *n* = 171 (%)
Sarcoma	184 (35.3)	64 (37.4)
Colorectal	86 (16.5)	27 (15.8)
Head & neck	37 (7.1)	9 (5.3)
Gynecologic	21 (4.0)	5 (2.9)
Esophageal	5 (1.0)	1 (0.6)
Renal	70 (13.4)	24 (14.0)
Urothelial	10 (1.9)	4 (2.3)
Melanoma	36 (6.9)	14 (8.2)
Germ cell	17 (3.3)	2 (1.2)
Cholangiocellular carcinoma	6 (1.2)	5 (2.9)
Pancreatic	5 (1.0)	2 (1.2)
Gastric	4 (0.8)	2 (1.2)
Hepatocellular carcinoma	7 (1.3)	0 (0.0)
Other	33 (6.3)	12 (7.0)

**Table 5:** Postoperative morbidity and mortality for the study groups.

Variable	Control *n* = 521 (%)	COPD *n* = 171 (%)	*p*-Value
At least one complication/patient	76 (14.6%)	33 (19.3%)	0.148
Air leak > 5 days	12 (2.3%)	15 (8.8%)	<0.001
ARDS	1 (0.2%)	1 (0.6%)	0.433
Postop. atelectasis req. intervention	4 (0.8%)	0 (0.0%)	0.577
Atrial arrhythmia	6 (1.2%)	2 (1.2%)	1.000
Ventricular arrhythmia	1 (0.2%)	0	1.000
Myocardial infarction	1 (0.2%)	0	1.000
Pneumonia	1 (0.2%)	0	1.000
Re-operation for bleeding	3 (0.6%)	1 (0.6%)	1.000
Chylothorax	1 (0.2%)	0	1.000
Renal failure	2 (0.4%)	2 (1.2%)	0.257
Wound infection	2 (0.4%)	0	1.000
30-day mortality	9 (1.7%)	0 (0.0%)	0.122
90-day mortality	12 (2.3%)	6 (3.5%)	0.409

**Table 6:** Baseline oncological and surgical characteristics of patients with COPD, stratified by severity (GOLD 1–2 vs. GOLD 3–4).

Variable	COPD GOLD 1–2 *n* = 126 (%)	COPD GOLD 3–4 *n* = 45 (%)	*p*-Value
Age, years, median	65	61	0.178
Primary tumour type			
Sarcoma	48 (38.1)	16 (35.6)	0.858
Colorectal	16 (12.7)	11 (24.4)	0.093
Renal cell	18 (14.3)	6 (13.3)	1.000
Melanoma	10 (7.9)	4 (8.9)	0.762
Other	34 (27.0)	8 (17.8)	0.313
Number of metastases, median	2	2	0.737
Unilateral disease	121 (96.0)	44 (97.8)	1.000
Bilateral disease	5 (4.0)	1 (2.2)	
VATS approach	50 (39.7)	26 (57.8)	0.054
Major resection	59 (46.8)	7 (15.6)	<0.001
Preoperative systemic therapy	29 (23.0)	3 (6.7)	0.015
Repeat pulmonary metastasectomy (RPM)	25 (19.8)	4 (8.9)	0.109

**Table 7:** Independent factors for postoperative morbidity in patients undergoing pulmonary metastasectomy.

Variable	Univariate OR (95% CI)	*p*-Value	Multivariate OR (95% CI)	*p*-Value
COPD	1.40 (0.89–2.20)	0.144	1.22 (0.76–1.94)	0.405
COPD GOLD severity (per grade)	1.20 (1.00–1.44)	0.045	1.18 (0.98–1.43)	0.079
Age (per year)	1.00 (0.99–1.02)	0.881	–	–
Male gender	0.84 (0.56–1.27)	0.403	–	–
Obesity (BMI > 30)	0.51 (0.25–1.04)	0.063	–	–
ASA ≥ 3	1.22 (0.71–2.10)	0.475	–	–
ECOG ≥ 1	2.26 (1.40–3.64)	0.001	2.09 (1.28–3.41)	0.003
At least one comorbidity	0.97 (0.56–1.69)	0.919	–	–
Arterial hypertension	1.14 (0.75–1.71)	0.541	–	–
Cardiovascular disease	1.20 (0.73–1.98)	0.472	–	–
Chronic kidney disease	0.19 (0.03–1.42)	0.106	–	–
Ever smoker	1.32 (0.88–1.99)	0.182	–	–
Surgical approach (VATS vs. THT)	0.53 (0.34–0.81)	0.003	0.58 (0.37–0.89)	0.013
Lymphadenectomy	0.91 (0.61–1.38)	0.664	–	–
Multiple metastases (≥2)	1.34 (0.76–2.36)	0.318	–	–
Repeat metastasectomy (RPM)	2.20 (1.26–3.85)	0.006	1.88 (1.06–3.35)	0.031
preoperative systemic therapy	0.94 (0.53–1.68)	0.840	–	–

**Table 8:** Independent factors for survival for patients undergoing pulmonary metastasectomy.

Variable	Univariate HR (95% CI)	*p*-Value	Multivariate HR (95% CI)	*p*-Value
COPD	1.13 (0.79–1.63)	0.496	1.01 (0.70–1.46)	0.953
COPD GOLD severity (per grade)	1.01 (0.86–1.18)	0.917	0.98 (0.83–1.16)	0.831
Age (per year)	1.02 (1.01–1.03)	0.002	1.02 (1.01–1.03)	0.003
Male gender	0.96 (0.70–1.33)	0.820	–	–
Obesity (BMI > 30)	0.74 (0.44–1.24)	0.254	–	–
ASA ≥ 3	1.33 (0.88–2.00)	0.175	–	–
ECOG ≥ 1	1.37 (0.91–2.05)	0.130	–	–
At least one comorbidity preop.	1.06 (0.68–1.66)	0.790	–	–
Arterial hypertension	1.35 (0.98–1.86)	0.065	–	–
Cardiovascular disease	1.02 (0.69–1.51)	0.907	–	–
Chronic kidney disease	1.19 (0.56–2.54)	0.655	–	–
Ever smoker	1.22 (0.88–1.67)	0.229	–	–
DFI	1.25 (0.85–1.84)	0.27		
DFS	1.30 (0.88–1.92)	0.19		
Surgical approach (VATS vs. THT)	0.62 (0.44–0.86)	0.004	0.74 (0.53–1.03)	0.075
Multiple metastases (≥2)	1.35 (0.86–2.10)	0.188	–	–
Repeat metastasectomy (RPM)	1.94 (1.25–3.00)	0.003	1.60 (1.03–2.50)	0.036
preoperative systemic therapy	2.29 (1.58–3.32)	<0.001	2.18 (1.49–3.20)	<0.001
Histology—colorectal	0.81 (0.49–1.34)	0.414	–	–
Histology—renal cell	0.87 (0.52–1.44)	0.579	–	–
Histology—melanoma	0.85 (0.44–1.67)	0.647	–	–
Histology—other (ref. sarcoma)	1.03 (0.70–1.53)	0.864	–	–

**Table 9:** Propensity Score-Matched Analysis (COPD vs. control group). Baseline characteristics in the matched cohort.

Variable	Control *n* = 170 (%)	COPD *n* = 170 (%)	*p*-Value	SMD
Age, median (years)	64	64	0.685	0.005
Male sex	96 (56.5)	96 (56.5)	1.000	0.000
Obesity (BMI > 30)	17 (10.0)	22 (12.9)	0.496	0.092
ASA ≥ 3	37 (21.8)	34 (20.0)	0.790	−0.043
ECOG ≥ 1	38 (22.4)	38 (22.4)	1.000	0.000
At least one comorbidity	157 (92.4)	154 (90.6)	0.698	−0.063
Arterial hypertension	94 (55.3)	86 (50.6)	0.447	−0.094
Cardiovascular disease	32 (18.8)	33 (19.4)	1.000	0.015
Chronic kidney disease	7 (4.1)	4 (2.4)	0.542	−0.100
Ever smoker	110 (64.7)	111 (65.3)	1.000	0.012
Multiple metastases (≥2)	143 (84.1)	138 (81.2)	0.567	−0.077
preoperative systemic therapy	31 (18.2)	32 (18.8)	1.000	0.015
Major resection	68 (40.0)	65 (38.2)	0.824	−0.036

**Table 10:** Univariable and multivariable Cox regression analysis of factors associated with overall survival in the propensity-matched cohort.

Variable	Univariate HR (95% CI)	*p*	Multivariate HR (95% CI)	*p*
COPD	1.05 (0.67–1.65)	0.835	–	–
Age (per year)	1.03 (1.01–1.05)	0.008	1.03 (1.01–1.06)	0.006
Male sex	1.03 (0.65–1.63)	0.888	–	–
Obesity (BMI > 30)	0.97 (0.45–2.13)	0.948	–	–
ASA ≥ 3	1.12 (0.65–1.92)	0.682	–	–
ECOG ≥ 1	1.24 (0.73–2.10)	0.433	–	–
At least one comorbidity	0.62 (0.31–1.24)	0.173	–	–
Arterial hypertension	1.29 (0.81–2.03)	0.282	–	–
Cardiovascular disease	1.31 (0.78–2.21)	0.305	–	–
Chronic kidney disease	1.50 (0.47–4.76)	0.492	–	–
Ever smoker	1.09 (0.68–1.77)	0.711	–	–
Surgical approach	0.57 (0.36–0.91)	0.020	0.70 (0.43–1.15)	0.158
Number of resected metastases	1.32 (0.70–2.50)	0.395	–	–
Repeat metastasectomy (RPM)	1.66 (0.91–3.02)	0.096	–	–
preoperative systemic therapy	2.38 (1.44–3.94)	0.001	2.31 (1.37–3.91)	0.002

## Data Availability

The data presented in this study are available upon request from the corresponding author.

## Abbreviations

The following abbreviations are used in this manuscript:

ABE	GOLD A, B, E assessment groups
ARDS	acute respiratory distress syndrome
ASA	American Society of Anesthesiologists
BMI	body mass index
CI	confidence interval
COPD	chronic obstructive pulmonary disease
DFI	disease-free interval
DFS	disease-free survival
DLCO	diffusing capacity of the lung for carbon monoxide
ECOG	Eastern Cooperative Oncology Group
FEV1	forced expiratory volume in 1 s
FVC	forced vital capacity
GOLD	Global Initiative for Chronic Obstructive Lung Disease
HR	hazard ratio
NSCLC	non-small cell lung cancer
OR	odds ratio
OS	overall survival
PM	pulmonary metastasectomy
R0	complete resection with microscopically negative margins
RPM	repeat pulmonary metastasectomy
SD	standard deviation
SMD	standardized mean difference
SPSS	Statistical Package for the Social Sciences (IBM SPSS Statistics)
VATS	video-assisted thoracoscopic surgery

## References

[1] Grapatsas K., Papaporfyriou A., Leivaditis V., Ehle B., Galanis M. (2022). Lung metastatectomy: Can laser-assisted surgery make a difference?. Curr. Oncol..

[2] Osei-Agyemang T., Palade E., Haderthauer J., Ploenes T., Yaneva V., Passlick B. (2013). Pulmonary metastasectomy: An analysis of technical and oncological outcomes in 301 patients with a focus on laser resection. Zentralbl. Chir..

[3] Pfannschmidt J., Egerer G., Bischof M., Thomas M., Dienemann H. (2012). Surgical intervention for pulmonary metastases. Dtsch. Arztebl. Int..

[4] Negewo N., McDonald V., Gibson P. (2015). Comorbidity in chronic obstructive pulmonary disease. Respir. Investig..

[5] Sekine Y., Iwata T., Chiyo M., Yasufuku K., Motohashi S., Yoshida S., Suzuki M., Iizasa T., Saitoh Y., Fujisawa T. (2003). Minimal alteration of pulmonary function after lobectomy in lung cancer patients with chronic obstructive pulmonary disease. Ann. Thorac. Surg..

[6] Iwasaki A., Shirakusa T., Enatsu S., Maekawa S., Yoshida Y., Yoshinaga Y. (2005). Surgical treatment for lung cancer with COPD based on the Global Initiative for Chronic Obstructive Lung Disease (GOLD). Thorac. Cardiovasc. Surg..

[7] Geldmacher H., Biller H., Herbst A., Urbanski K., Allison M., Buist A., Hohlfeld J., Welte T. (2008). The prevalence of chronic obstructive pulmonary disease (COPD) in Germany. Results of the BOLD study. Dtsch. Med. Wochenschr..

[8] Mirza S., Clay R.D., Koslow M.A., Scanlon P.D. (2018). COPD Guidelines: A Review of the 2018 GOLD Report. Mayo Clin. Proc..

[9] Sharma M., Joshi S., Banjade P., Ghamande S.A., Surani S. (2024). Global Initiative for Chronic Obstructive Lung Disease (GOLD) 2023 Guidelines Reviewed. Open Respir. Med. J..

[10] Abbott T.E.F., Fowler A.J., Pelosi P., Gama de Abreu M., Møller A.M., Canet J., Creagh-Brown B., Mythen M., Gin T., Lalu M.M. (2018). A systematic review and consensus definitions for standardised end-points in perioperative medicine: Pulmonary complications. Br. J. Anaesth..

[11] Seely A., Ivanovic J., Threader J., Al-Hussaini Derar Al-Shehab A., Ramsay T., Gilbert S., Maziak D.E., Shamji F., Sundaresan R.S. (2010). Systematic classification of morbidity and mortality after thoracic surgery. Ann. Thorac. Surg..

[12] Kahnert K., Jörres R.A., Behr J., Welte T. (2023). The diagnosis and treatment of COPD and its comorbidities. The diagnosis and treatment of COPD and its comorbidities. Dtsch. Arztebl. Int..

[13] Greulich T., Weist B.J.D., Koczulla A.R., Janciauskiene S., Klemmer A., Lux W., Alter P., Vogelmeier C.F. (2017). Prevalence of comorbidities in COPD patients by disease severity in a German population. Respir. Med..

[14] Senbaklavaci O. (2014). Lobar Lung Resection in Elderly Patients with Non-Small Cell Lung Carcinoma: Impact of Chronic Obstructive Pulmonary Disease on Surgical Outcome. Int. Surg..

[15] Grapatsas K., Dörr F., Menghesha H., Schuler M., Grünwald V., Bauer S., Schmidt H.H.-J., Lang S., Kimmig R., Kasper S. (2023). New Prognostic Score (Essen Score) to Predict Postoperative Morbidity after Resection of Lung Metastases. Cancers.

[16] Grapatsas K., Hassan M., Semmelmann A., Ehle B., Passlick B., Schmid S., Le U.T. (2022). Should cardiovascular comorbidities be a contraindication for pulmonary metastasectomy?. J. Thorac. Dis..

[17] Hassan M., Ehle B., Passlick B., Grapatsas K. (2022). Lung Resections for Elderly Patients with Lung Metastases: A Comparative Study of the Postoperative Complications and Overall Survival. Curr. Oncol..

[18] Pastorino U., Buyse M., Friedel G., Ginsberg R.J., Girard P., Goldstraw P., Johnston M., McCormack P., Pass H., Putnam J.B. (1997). Long-term results of lung metastasectomy: Prognostic analyses based on 5206 cases. J. Thorac. Cardiovasc. Surg..

[19] Petrella F., Diotti C., Rimessi A., Spaggiari L. (2017). Pulmonary metastasectomy: An overview. J. Thorac. Dis..

[20] Lickerm M.J., Widikker I., Robert J., Frey J.G., Spiliopoulos A., Ellenberger C., Schweizer A., Tschopp J.M. (2006). Operative mortality and respiratory complications after lung resection for cancer: Impact of chronic obstructive pulmonary disease and time trends. Ann. Thorac. Surg..

[21] Sekine Y., Suzuki H., Yamada Y., Koh E., Yoshino I. (2013). Severity of chronic obstructive pulmonary disease and its relationship to lung cancer prognosis after surgical resection. Thorac. Cardiovasc. Surg..

[22] Rodríguez-Fuster A., Belda-Sanchis J., Aguiló R., Embun R., Mojal S., Call S., Molins L., de Andrés J.J.R. (2014). Morbidity and mortality in a large series of surgical patients with pulmonary metastases of colorectal carcinoma: A prospective multicentre Spanish study (GECMP-CCR-SEPAR). Eur. J. Cardiothorac. Surg..

[23] Sponholz S., Schirren M., Oguzhan S., Schirren J. (2018). Morbidity, mortality, and survival in elderly patients undergoing pulmonary metastasectomy for colorectal cancer. Int. J. Color. Dis..

[24] Lin H., Lu Y., Lin L., Meng K., Fan J. (2019). Does chronic obstructive pulmonary disease relate to poor prognosis in patients with lung cancer?: A meta-analysis. Medicine.

[25] Xu W., Zhu J., Li L., Li D., Du R. (2022). The Prognostic Role of Chronic Obstructive Pulmonary Disease for Lung Cancer After Pulmonary Resection. J. Surg. Res..

[26] Schmid S., Minnella E.M., Pilon Y., Rokah M., Rayes R., Najmeh S., Cools-Lartigue J., Ferri L., Mulder D., Sirois D. (2022). Neoadjuvant Prehabilitation Therapy for Locally Advanced Non-Small-Cell Lung Cancer: Optimizing Outcomes Throughout the Trajectory of Care. Clin. Lung Cancer.

[27] Downey R.J. (1999). Surgical treatment of pulmonary metastases. Surg. Oncol. Clin. N. Am..

[28] Treasure T. (2008). Pulmonary metastasectomy for colorectal cancer: Weak evidence and no randomised trials. Eur. J. Cardi-Othorac. Surg..

[29] Welter S., Jacobs J., Krbek T., Krebs B., Stamatis G. (2007). Long-term survival after repeated resection of pulmonary metas-tases from colorectal cancer. Ann. Thorac. Surg..

[30] Molnar T.F., Gebitekin C., Turna A. (2010). What are the considerations in the surgical approach in pulmonary metas-tasectomy?. J. Thorac. Oncol..

[31] Abdelnour-Berchtold E., Perentes J.Y., Ris H.B., Beigelman C., Lovis A., Peters S., Krueger T., Gonzalez M. (2016). Survival and local recurrence after video-assisted thoracoscopic lung metastasectomy. World J. Surg..

